# DESIGN, RECRUITMENT, AND BASELINE CHARACTERISTICS OF THE AGING AND COGNITIVE HEALTH EVALUATION IN ELDERS TRIAL

**DOI:** 10.1093/geroni/igad104.0296

**Published:** 2023-12-21

**Authors:** Nicholas Reed

**Affiliations:** Johns Hopkins Bloomberg School of Public Health, Baltimore, Maryland, United States

## Abstract

Hearing loss is highly prevalent among older adults and independently associated with cognitive decline. This presentation will describe the design and baseline characteristics of the first randomized controlled trial to test whether hearing treatment can reduce cognitive decline, the Aging and Cognitive Health Evaluation in Elders (ACHIEVE) trial. This multicenter study was nested within the Atherosclerosis Risk in Communities (ARIC) observational cohort study. The primary outcome is 3-year change in a global cognitive function factor score derived from a comprehensive cognitive test battery. Other prespecified outcomes include loneliness, social network size, depressive symptomatology, health-related quality of life, hospitalization, falls, physical function, and accelerometry-measured physical activity. Cognitively intact, community-dwelling 70-84-year-old participants with adult-onset hearing loss were recruited from the parent ARIC study and de novo from the surrounding communities and randomized 1:1 to the best practices hearing intervention or successful aging health education control intervention. Over a 24-month period, 3004 telephone screenings resulted in 2344 in-person hearing, vision, and cognition screenings and 1294 comprehensive screenings. Among 1102 eligible, 977 were randomized into the trial (median age = 76.4; 53.5% female; 87.6% White; 53.3% held a bachelor’s degree or higher). Participants from the parent ARIC study were recruited much earlier in the process and were less likely to report hearing loss that interfered with their quality of life relative to de novo participants recruited from the community. Minor differences in baseline hearing or health characteristics were found by recruitment method (i.e., ARIC or de novo) or by study site.

